# Is there progress in the adsorbent development for water treatment?

**DOI:** 10.1016/j.isci.2025.112993

**Published:** 2025-06-26

**Authors:** Tero Luukkonen, Juhani Teeriniemi

**Affiliations:** 1 Fibre and Particle Engineering Research Unit, University of Oulu, P.O. Box 4300, 90014 Oulu, Finland; 2 Department of Chemical Engineering Technology, University of Johannesburg, P.O Box 17011, Doornfontein 2088, South Africa; 3 AI4Value Oy, Kampinkuja 2, 00100 Helsinki, Finland

**Keywords:** Applied sciences, Water resources engineering, Engineering

## Abstract

Adsorption technology is an essential part of modern water treatment. However, only a handful of adsorbent materials are used industrially, which is in striking contrast with the exponentially increasing scientific publishing activity. In this perspective, the aim is to discuss the global progress on the adsorbent materials development by using the reported adsorption capacities as a simple probe. An automated text analyzer employing a large language model was used to extract adsorption data. Total 11,664 scientific journal articles about ammonium, arsenic, lead, methylene blue, or nitrate adsorption covering years 1973–2023 were analyzed. When the adsorption capacities were plotted as a function of the publication year, a two-fold trend was revealed: most of the studies (i.e., up to the 50–75% percentile ranks) revealed none or only modest improvement in the adsorption capacities, while the very best materials exhibited staggering capacities up to the order of 10^3^–10^4^ mg g^−1^.

## Graphical abstract

**Figure:**
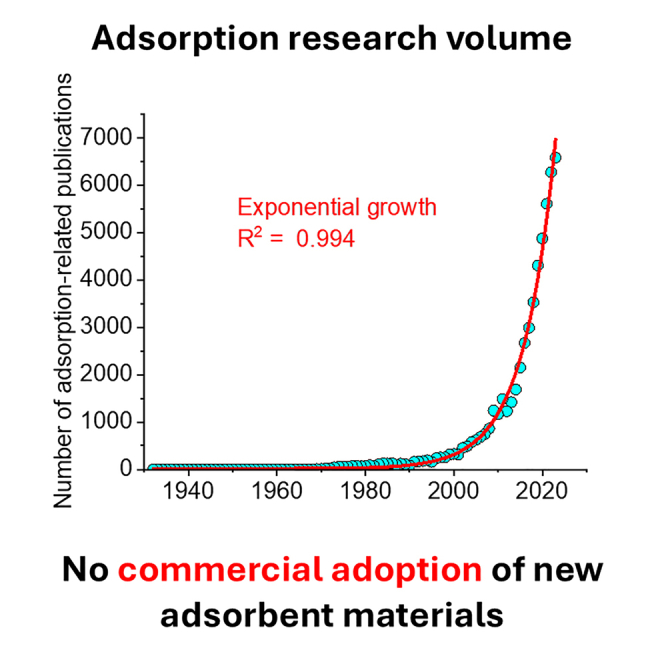

## Status of adsorption technology in water treatment

Adsorption technology is an important part of modern water treatment. However, only few adsorbent materials are widely used on an industrial scale. The most commonly used material, activated carbon, has been employed for the removal of color and taste from drinking water since the 1920s and for the tertiary wastewater treatment since the 1960s.^1^ In recent years, activated carbon has been implemented for the separation of organic micropollutants from municipal wastewater in full-scale processes due to new legislative requirements, for example, in Switzerland.^2^^,^^3^^,^^4^ Ion exchange resins (i.e., cross-linked polymer networks containing bonded ion-exchange ligands) were introduced for the commercial use in the 1940s, for example, to the demineralization of water for boiler plants and other industries.^5^ Zeolites have been applied for water softening for more than 120 years and their other potential water and wastewater treatment applications (e.g., ammonium removal) have been widely acknowledged.^6^ Nevertheless, the use of zeolites in full-scale wastewater treatment plants has remained limited—as far as the authors are aware, there is only one municipal wastewater treatment plant (Tahoe-Truckee Sanitation Agency, CA, USA, with a treatment capacity of 26,100 m^3^/d) globally that is using natural zeolite for ammonium separation.^7^ Additional examples of adsorbents used commercially (either full-scale treatment plants or point-of-use filters) include activated alumina and hydrous ferric oxide for removal of anions such as arsenic, fluoride, or phosphate.^8^^,^^9^

However, outside of the above materials, not many others are used industrially in the water or wastewater treatment sectors. This is in a stark discrepancy with the publication volume of the scientific articles about the development and testing of new potential adsorbents which has been increasing exponentially during the last 20 years. For example, Wolkersdorfer notes in his book on mine water treatment that “*despite extensive search, I have not been able to find an industrial-scale plant that treats mine water using artificial sorbents*” (though he mentions that there is one operational plant using iron hydroxide to remove arsenic).^10^ This highlights the peculiar bias in the adsorption literature, which is that many articles state that adsorption processes are commonly *used* for wastewater treatment (implicating full-scale implementation) while they are in fact commonly *studied* but not commonly used in practice. Recently, the following obstacles for the commercialization of new adsorbents were identified by Kunwar et al.^11^: (1) slowness and high costs caused by regulation governing the types of materials that can be used; (2) treatment costs should be lower than with the existing adsorbents; (3) concerns that the treatment performance would be compromised with novel materials; and (4) reluctance to change the current adsorbent suppliers and try out new materials. Also, Roberts^12^ emphasizes that when innovations are implemented in water or wastewater treatment, they need to be necessary, cost-effective, and low-risk. Thus, there appears to be several non-technical barriers for the introduction of new adsorbents for industrial use.

There are widely spread methodological problems in adsorption-related scientific research. It has been questioned whether by conducting adsorption studies in highly idealized conditions (i.e., batch experiments with synthetic wasterwater, that is, adsorbates dissolved in pure water), any practically meaningful new knowledge is created.^10^ Additionally, there are various misconceptions, misuses, and misinterpretations of the mathematical models used to describe the adsorption phenomena (e.g., breakthrough curves in flow-through column experiments, adsorption equilibria in batch experiments, or kinetics). Some frequently occurring problems in the articles include wrong mathematical forms of the equations due to the faulty (secondary) referencing,^13^ incorrect linearization of equations (e.g., the same variable can be represented as both dependent and independent),^14^^,^^15^ wrong or lacking use of units for parameters,^16^ or mechanism interpretation based on an empirical equation fitting or assuming causation from correlation.^17^^,^^18^ In terms of the adsorption kinetics, the use of the reaction kinetic models (i.e., the (pseudo-)first-order or pseudo-second-order rate laws) is deemed appropriate only for weakly porous adsorbents with chemisorption as the major mechanism and without film-diffusion as the limiting factor.^19^ Thus, their use is discouraged.^19^^,^^20^ Moreover, it is very common to declare novel adsorbents “low-cost”, “sustainable”, or “green” without conducting any life cycle or economic analyses. Interestingly, a whole genre within the adsorption-related research literature has emerged that focuses on identifying the earlier errors and mistakes^21^ or providing guidance on how to avoid them.^22^

## Probing the progress in adsorbent material development

As a result of the lack of commercial implementation of novel adsorbents and various problems in the research, one may wonder whether any real progress in the adsorbent development has been made despite the immense increase in the research activity, which also means spending significant resources on the topic. In the present perspective article, we aimed to answer this question by probing the global adsorbent development by collecting an extensive dataset of adsorption capacities of selected adsorbates and analyzing how they have evolved over time. The adsorption capacity expresses the maximum amount of an adsorbate that an adsorbent can uptake at equilibrium under the experimental conditions and it is a major parameter determining the sustainability (especially from an economical viewpoint) of an adsorbent.^23^ Thus, it is a good and simple numerical probe for the purpose of this study.

**Figure 1:**
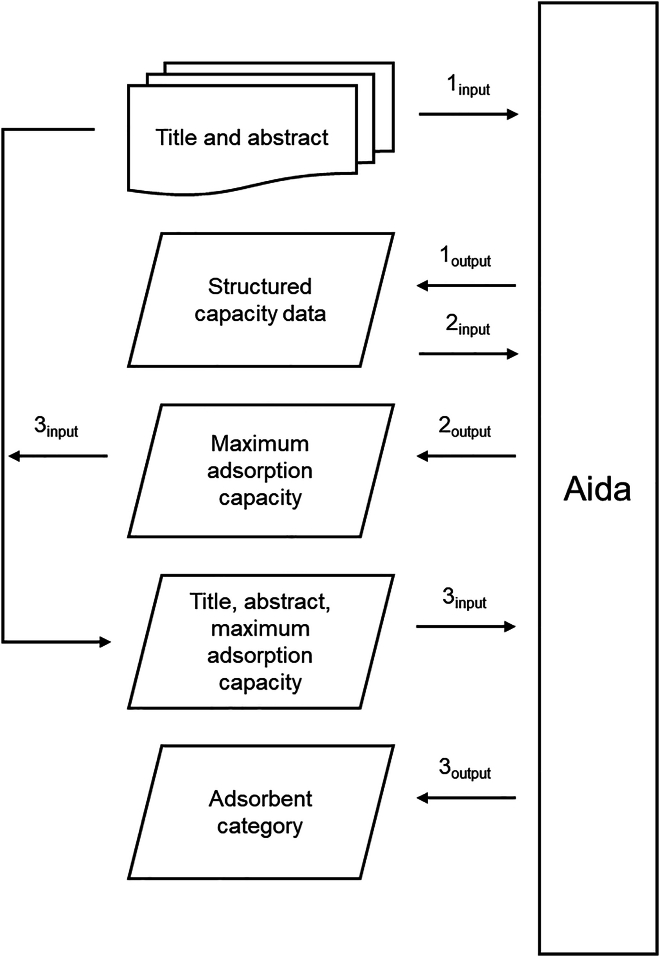
Process of automated data extraction with Aida The process consists of three steps to extract both the reported maximum adsorption capacity and the category of adsorbent material. The steps are divided into input and output parts. First, the information for each material was extracted from the abstract into a structured format containing the name of the adsorbent, name of the adsorbate, adsorption capacity, and unit. If several materials were mentioned, only the material with the highest capacity was selected. The categories of the best adsorbent materials were deduced from the abstract into predefined 23 different classes (i.e., carbon nanotubes, biomass/biopolymers, metal oxides, metal-organic frameworks, metal hydroxides, hydrogels, other natural minerals, graphene oxides, covalent organic frameworks, metal carbonates, biochars, zeolites, clay minerals, Mxenes, other aluminosilicates, hydroxyapatites, other unclassified, other organic polymers, commercial ion-exchange resins, Mo sulfides, activated carbons, layered double hydroxides, and silica).

The data collection was conducted by searching the Scopus database, exporting the results as a spreadsheet file, and then harnessing a large language model (LLM), AI Document Assistant (Aida, developed by AI4Value, Finland) employing GPT 3.5 turbo,^24^ to retrieve the adsorption capacities mentioned in the article abstracts. Paywalls of scientific publishers were avoided by focusing on freely available abstracts. Of course, not all studies mention the observed adsorption capacity in the abstract but, nevertheless, total 11,664 values could be retrieved with this method. Figure 1 shows the schematic process of the automated data extraction with Aida. More details on the methodology and the spreadsheets are available in the Fairdata data repository.^25^ The selected adsorbates were ammonium, nitrate, arsenic, methylene blue, and lead as they represent diverse characteristics (e.g., ionic charge and aqueous radius). The adsorption of these species from water has been actively studied due to their toxicity (arsenic and lead), eutrophication potential (ammonium and nitrate), or convenience as an adsorption efficiency indicator (methylene blue, commonly reported as methylene blue number for activated carbons). The reliability of the data collection was verified by selecting randomly 100 studies from the spreadsheet and manually finding the maximum adsorption capacities from their abstracts. When comparing the results to the ones obtained by Aida, 98% of the results were similar. In the 2%, the interpretation of the abstract in terms of the reported maximum adsorption capacity was not unambiguous (e.g., there were several numbers mentioned and the sentence structure was unclear). Based on this validation, the results can be considered very accurate for statistical analyses.

The approach of using LLMs for data retrieval in the context of adsorption research is novel as no similar studies have been published. Surprisingly, also, the use of systematic literature review and meta-analysis methodology for adsorption literature is rather uncommon: if searching the Scopus database with a search phrase *adsorption AND “meta-analysis”*, only 20 articles with a clear relevance to water or wastewater treatment can be found.^26^^,^^27^^,^^28^^,^^29^^,^^30^^,^^31^^,^^32^^,^^33^^,^^34^^,^^35^^,^^36^^,^^37^^,^^38^^,^^39^^,^^40^^,^^41^^,^^42^^,^^43^^,^^44^^,^^45^ Based on the vast adsorption literature, there could certainly be more meta-analyses as they provide more comprehensive understanding about the state of the art than common narrative reviews. In that sense, the present paper aims to provide quantitative information on the performance evolution trend of adsorbents.

## Evolution of adsorption capacities over time and identifying the top materials

**Figure 2:**
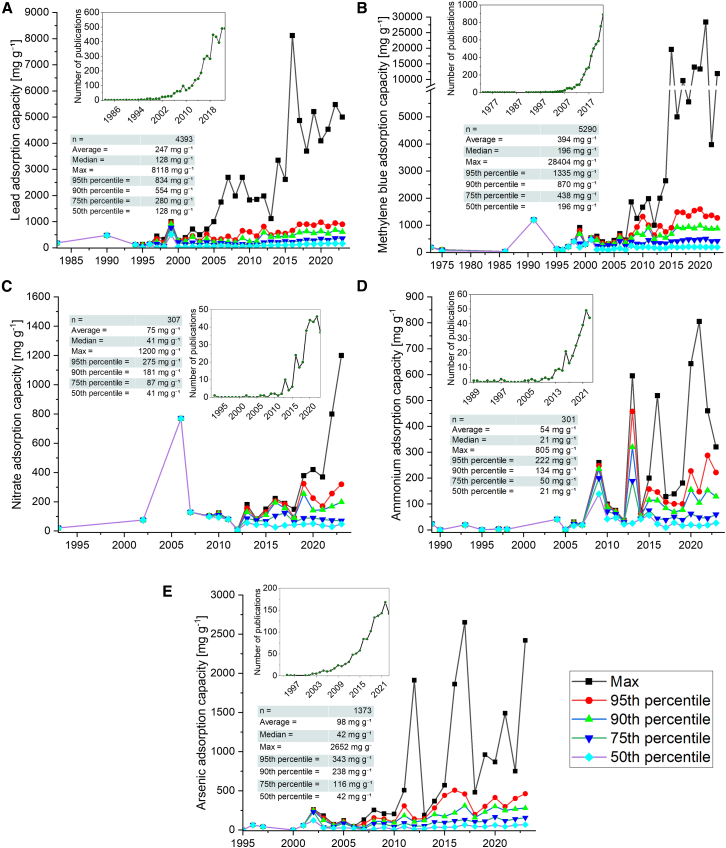
Overview of the adsorption capacity development over time per publication year The analyzed adsorbates are: (A) lead, (B) methylene blue, (C) nitrate, (D) ammonium, and (E) arsenic shown as 50–95% percentile ranks with maximum values. The insets show the evolution of annual publication volume and statistical parameters of the whole dataset per adsorbate. Full list of references used to construct this figure can be found from Luukkonen and Teeriniemi.^25^

Figure 2 shows the evolution of adsorption capacities of lead, methylene blue, nitrate, ammonium, and arsenic over time. The data reveals that the publication volume on each of the adsorbates (shown in the insets of Figure 2) began to accelerate during the early 2000s, and thus we focus on that time frame. As a general trend, the rate of adsorption capacity increase decreases drastically as going down in the percentile ranks from 95% to 75%. For ammonium and nitrate, there is in practice no development in the adsorption capacities when considering 75% of the analyzed literature (i.e., the 75% percentile rank). For arsenic, lead, and methylene blue, there is still an increasing trend (albeit modest) in the adsorption capacities when considering the 75% and 50% percentile ranks (for arsenic, the development diminishes at 50% percentile rank). Nevertheless, the strong increase in the publication volume appears to correlate poorly with the evolution of adsorption capacities. However, the highest reported adsorption capacities per publication year appear to increase more closely hand in hand with the publication volume. Thus, it appears that a large proportion of the adsorption studies always arrive to closely similar results in terms of the adsorption capacity while the best materials are exhibiting tremendous progress. The data in Figure 2 can be also used to assess the state-of-the-art performance of adsorbent materials for the selected adsorbates. For example, in order to perform better than 75% of the published studies, the adsorption capacity of methylene blue should be 438 mg g^−1^ (Figure 2B).

**Figure 3:**
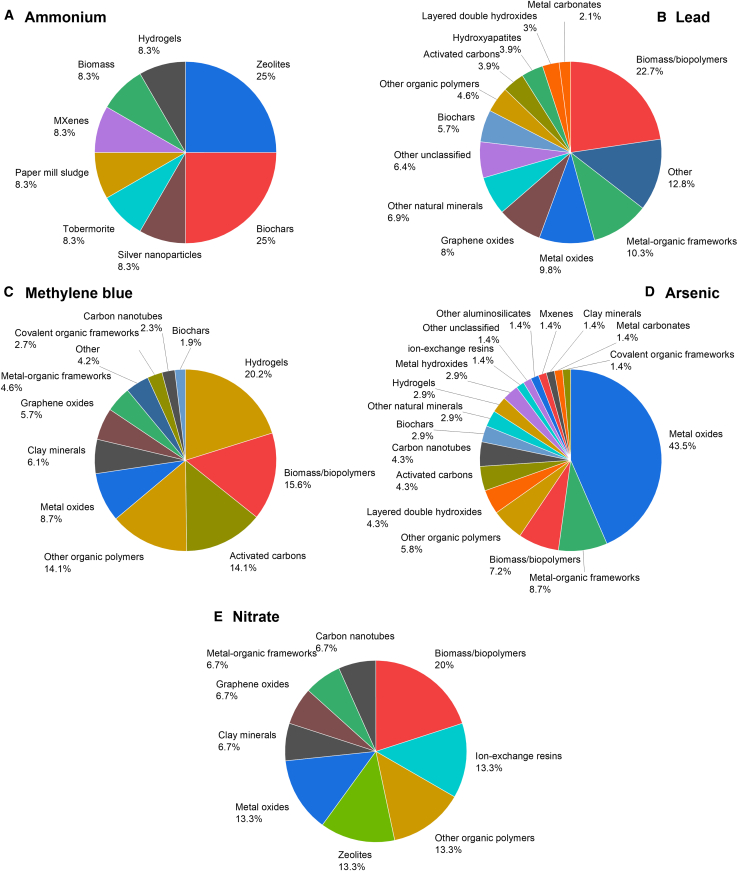
The adsorbent material classes representing the top 5% performance The analyzed adsorbates are: (A) ammonium (q ≥ 222 mg g^−1^), (B) lead (q ≥ 834 mg g^−1^), (C) methylene blue (q ≥ 1335 mg g^−1^), (D) arsenic (q ≥ 343 mg g^−1^), and (E) nitrate (q ≥ 275 mg g^−1^).

To analyze further the best-performing adsorbent types, we focus on the top 5% of the adsorbents (i.e., those above the 95% percentile rank in Figure 2): their distribution into different material classes and the top five adsorbents for each adsorbate are shown in Figure 3 and Table 1, respectively. The highest-performing ammonium adsorbents (Figure 3A) are dominated by surprisingly conventional materials: zeolites and biochars. The best adsorbent for ammonium is a biochar based on date palm waste which could reportedly reach ∼800 mg g^−1^ capacity.^46^ Regardless of this promising performance, biochar is not used in full-scale wastewater treatment for ammonium removal. A 70-day bench-scale study has been reported in which quartz sand and *Miscanthus* biochar were compared as filtration media of raw municipal wastewater pre-treated by an anaerobic pond.^71^ The results demonstrated that sand and biochar were equally ineffective at removing ammonium nitrogen (<7% removal). Thus, the selectivity or surface clogging could be an issue if aiming to use biochar for ammonium removal in realistic wastewater treatment conditions. Also, with nitrate (Figure 3E), the best adsorbents were conventional materials: commercial anion exchange resins or modified clays/zeolites. The highest capacity, ∼1200 mg g^−1^, was reported for an unspecified strong base anion exchanger resin.^66^ With lead and methylene blue, the best adsorbents were various engineered nanomaterials (e.g., carbon nanotubes, graphene oxides, metal-organic frameworks, or MXenes) which have reportedly reached up to ∼8100 mg g^−1^ and ∼28 000 mg g^−1^ capacities, respectively.^51^^,^^56^ With arsenic, metal oxides represented the largest high-performing material category (Figure 3D) but several of the best materials employed biosorption and bioaccumulation with bacteria biofilms (Table 1): *Bacillus arsenicus* MTCC 4380 biofilm supported on sawdust/MnFe_2_O_4_ composite could reportedly reach ∼2600 mg g^−1^ capacity.^61^ In general, most of the highly performing adsorbent materials had some form (and frequently rather complicated) chemical surface modification to enhance their performance or they were hybrid/composite materials.

**Table 1:** The best five adsorbents for each of the selected adsorbates

Adsorbate	Category	Adsorbent	Modification	Capacity [mg g^−1^]	References
NH_4_^+^	Biochars	Date palm waste	–	805	Fseha et al.^46^
NH_4_^+^	Zeolites	NaY zeolite	Inclusion of amorphous carbon	642	Shui et al.^47^
NH_4_^+^	Biomass	Peat	–	595	Kučić et al.^48^
NH_4_^+^	Biochars	Agricultural waste	Alkali modification	519	Liu et al.^49^
NH_4_^+^	MXenes	Ti_3_C_2_T_x_ MXene	–	460	Mansoor et al.^50^
Pb	Carbon nanotubes	Carbon nanotubes	Inclusion of polyrhodanine	8118	Alizadeh et al.^51^
Pb	Biopolymers	Tragacanth	Oxidation and reaction with L-arginine	5482	Saren et al.^52^
Pb	Metal oxides	Spherical MgO	–	5214	Kuang et al.^53^
Pb	Graphene oxides	Graphene oxide	Combination with zeolitic imidazolate framework	5000	Al-Nowaiser et al.^54^
Pb	Carbon nanotubes	Carbon nanotubes	Poly-amidoamine dendrimers	4870	Hayati et al.^55^
MB	MXenes	MXene	Inclusion of single-walled carbon nanotubes	28404	Yao et al.^56^
MB	Metal oxides	Ni molybdate	–	16863	Rakass et al.^57^
MB	MOFs	Co MOF with morpholine	–	14721	Ahmad et al.^58^
MB	MOFs	Co/C microrods	–	13960	Ye et al.^59^
MB	Hydrogels	Graphene-like carbon	Addition of PVA and freeze-drying	13382	Yang et al.^60^
As	Biomass/biopolymers	Sawdust/MnFe_2_O_4_	*Bacillus arsenicus* MTCC 4380 biofilm	2652	Podder and Majumder^61^
As	Biomass/biopolymers	*Pseudomonas* sp. SMS11	–	2422	Xiaoman et al.^62^
As	Hydrogels	Polymerized AMPS and 3-acrlylamidopropil-trimethyl	–	2336	Bakyt et al.^63^
As	Activated carbons	Granular activated carbon	Fe impregnation	1912	Chang et al.^64^
As	Metal oxides	Sawdust/MnFe_2_O_4_	*Corynebacterium glutamicum* MTCC 2745	1862	Podder and Majumder^65^
NO_3_^−^	Ion-exchange resins	Unspecified strong base anion exchange resin	–	1200	Yaragal and Mutnuri^66^
NO_3_^−^	Clay minerals	Montmorillonite	Cetylpyridinium and microcrystalline cellulose	800	Amaly et al.^67^
NO_3_^−^	Ion-exchange resins	Amberlite IRA 400	–	769	Chabani et al.^68^
NO_3_^−^	Zeolites	Unspecified zeolite	Chitosan and Fe^3+^ and alkaline treatment	498	Hidayat et al.^69^
NO_3_^−^	Zeolites	Clinoptilolite	γ-Fe_2_O_3_ nanoparticles	422	Darezereshki et al.^70^

MB, methylene blue; MOF, metal organic framework; PVA, polyvinyl alcohol; AMPS, 2-acrylamido-2-methylpropane sulfonic acid.

## Discussion

The best material classes identified by the present article contain several relatively new materials (Figure 3). For example, metal-organic frameworks were first introduced in 1995^72^ but only few commercial products based on them existed in 2024.^73^ Especially when considering adsorbents, the production of metal-organic frameworks on a sufficient scale and low enough cost level represents a bottleneck for their wide utilization.^73^ This likely applies also to other highly promising advanced materials shown in Figure 3 (e.g., graphene oxides, carbon nanotubes, or MXenes). Another important aspect is the environmental sustainability of the adsorbent material. Life cycle assessment (LCA) between conventional activated carbon produced from biomass via physical activation with carbon dioxide^74^ and zeolitic imidazolate framework-67 MOF produced with a solvothermal synthesis^75^ indicates that the material production causes surprisingly similar impacts (e.g., 18.8 and 14.1 kg CO_2_-eq global warming potential for activated carbon and MOF, respectively when producing 1 kg of the materials).

When considering wastewater treatment, there have been no strong incentives to use advanced treatment, such as adsorption technology. However, this might be now changing as the European Union directive on urban wastewater (EU 2024/3019) has been revised in November 2024 to include, for example, the requirement to remove organic micropollutants with quaternary treatment steps (i.e., 80% removal for indicator substances) at wastewater treatment plants with 150,000 population equivalent treatment capacity (or 10,000 population equivalent if the receiving water body is sensitive for pollution). Similar legislation exists in Switzerland since 2016 and the first wastewater treatment plants have been already upgraded to include additional treatment, such as dosing powdered activated carbon.^4^ Thus, adsorption technology could have an important role in achieving these targets. Another driver for the wider adoption of adsorption technology could be resource (e.g., nitrogen or phosphorus) recovery from wastewaters, in which an adsorption-desorption processes could be used as a separation and concentration method.^76^ Thus, new more stringent legislative requirements and changing the paradigm of *pollutant removal* to *resource recovery* could create markets for the new advanced adsorbents. Globally, the focus of the legislation governing wastewater treatment is on primary or secondary treatment and this should be improved.

Wolkersdorfer mentions in his book about a questionable practice related to the adsorption research: by varying the adsorbent material and synthetic wastewater composition and by determining the routine adsorption characteristics, it is possible to generate almost infinite number of publications with limited practical or industrial interest.^10^ He gives the following recommendation in the context of mine water treatment: “*If you are doing your own work on sorbents for mine water, please try to develop a process to incorporate the sorbents into a fixed bed, limit the competition of the different ions in the water, and treat a water stream larger than 1 L min*^*−1*^*. If not – just don’t do it. After all, with the right choice of sorbent and ion to be sorbed in synthetic mine water, you will always get a result anyway.*”^10^ This recommendation is easy to agree with. Such poor practices and emphasis on research quantity over quality could be additional reasons for the rather limited progress exhibited by the bulk of the analyzed studies in the present article.

How high should the adsorption capacity of an adsorbent be for commercial exploitation? Of the adsorbates discussed in the present article, ammonium and arsenic are removed from water using commercial adsorbents also in practice, at least to some extent. Thus, comparison of the commercially used materials to the trends reported in the present study might be useful. For ammonium adsorption, the Tahoe-Truckee Sanitation Agency wastewater treatment plant (CA, USA) uses a natural zeolite, clinoptilolite.^77^ The ammonium exchange capacity of clinoptilolite can be up to ∼25 mg g^−1^, but it is strongly affected, for example, by the purity of the mineral.^78^ If comparing that number to the ammonium adsorption capacity evolution (Figure 2D), it is slightly above the median value (i.e., 21 mg g^−1^, of 301 data points). Thus, any material capable of capturing at least 25 mg g^−1^ ammonium could be commercially interesting. On the other hand, this demonstrates that 50% of the capacities reported in Figure 2D are below the commercial benchmark value of clinoptilolite. However, the additional benefits of clinoptilolite is that it is selective for larger cations (in the following order: Cs > Rb > K > NH_4_^+^ > Ba > Sr > Na > Ca > Fe > Al > Mg > Li) and it is abundantly available (the estimated reserves of natural zeolites in the USA alone are estimated as > 80 Mt while the global production of zeolites was ∼1.1 Mt in 2023).^77^^,^^79^ For arsenic, granular activated alumina is a commercially used adsorbent in drinking water treatment and its capacity can be up to ∼3 mg g^−1^ for As(III) and ∼50 mg g^−1^ for As(V).^80^ Another commercial arsenic adsorbent, hydrous ferric oxide, can reach ∼5–10 mg g^−1^ adsorption capacity for As(V). From Figure 2E, showing the arsenic adsorption capacity evolution (without differentiating As(III) and As(V)), it can be observed that commercial activated alumina has higher capacity than 50% of the summarized materials (i.e., median of 42 mg g^−1^, of 1373 data points).

## Conclusions

An analysis of adsorption capacities from the literature was performed by utilizing an AI-assisted data collection. Using an LLM for tabulating numerical and textual data from the freely available article abstracts was very successful: 11,664 data points could be conveniently recovered, covering years 1973–2023. The 50% percentile rank of the analyzed literature represented only a very modest trend of development in the adsorption capacities. This suggests that a large proportion of the adsorption literature contributes rather little development when using adsorption capacity as a probe. The use of adsorption capacity as a single parameter to observe the adsorbent material development trend has limitations (e.g., there can be development in other areas such as material cost, reusability, or adsorption kinetics) but it is a central factor defining the operating expenses of an adsorption process. Second, it is a parameter reported very universally in almost every study assessing materials as adsorbents. Thus, we argue that the trends displayed in this article, with their limitations, are useful for providing a snapshot of the current state of the art on an unprecedented scale (summarizing 11,664 articles) and stirring up critical discussion. The very best adsorbents, in contrast to the bulk of the materials reported in the literature, displayed strong progress. The best adsorbents for lead, arsenic, and methylene blue comprise various engineered nanomaterials (e.g., carbon nanotubes, MXenes, or metal-organic frameworks). For ammonium and nitrate, the best adsorbents included surprisingly conventional materials (e.g., zeolites, biochars, or commercial ion exchange resins).

As recommendations based on this article, we propose the following for the researchers working on adsorption:

• Select the methodology (e.g., adsorption conditions) and models of adsorption equilibrium, kinetics, and dynamic column performance carefully and critically to be realistically representative. Avoid the common methodological pitfalls (there are plenty of guideline articles available). Interpret the results critically.
• Consult the previous literature: there are tens of thousands of publications related to adsorption. Evaluate whether your research really provides new information filling a knowledge gap. Place your results into the context of the state-of-the-art adsorbents.
• Create a road map of how the scientific results could be taken into practical use.

## Limitations of the study

One limitation of this study is that measuring the adsorbent development progress with a single numerical value, adsorption capacity, is a simplified approach. There are, of course, several other practically important factors that need to be also optimized and developed in addition to adsorption capacity (such as kinetics, regeneration, economics, or environmental sustainability). However, many adsorption studies, especially those aiming to develop new adsorbent materials, employ adsorption capacity as the central parameter. Especially, the studies which mention adsorption capacity in their article abstract obviously consider it as an important result. Second, adsorption capacity is a central factor in defining the operational expenses of an adsorption process in practice as it determines on how frequently the adsorbent needs to be regenerated or replaced. Thus, we argue that adsorption capacity is an appropriate single parameter to demonstrate the overall development in the adsorption technology field. It is also common practice in the research articles to compare the achieved adsorption capacity to the previous literature to demonstrate the progress. In that sense, the current study serves as a benchmark for global development in adsorption capacities and thus may help researchers to place their results in the context of existing literature. Another limitation of the present article is that the data are extracted from abstracts only and obviously not all publications report the adsorption capacity in the abstract. Nevertheless, we provide a snapshot of the current state of the art in adsorbent materials on an unprecedented scale (summarizing 11,664 articles).

## Acknowledgments

This work was supported by the funding received from the 10.13039/501100000780European Union’s Horizon Europe research and innovation program under the grant agreement no. 101058162 (AshCycle).

## Author contributions

T.L., conceptualization, validation, formal analysis, investigation, data curation, writing – original draft, visualization, project administration, and funding acquisition; J.T. methodology, software, validation, investigation, resources, and writing – review and editing.

## Declaration of interests

The authors declare no competing interests.

## Declaration of generative AI and AI-assisted technologies in the writing process

During the preparation of this work, the authors used Aida employing GPT 3.5 turbo in order to collect and tabulate adsorption-related information (e.g., adsorption capacity, adsorbate, and adsorbent type) from literature. After using this tool, the authors reviewed and edited the content as needed and take full responsibility for the content of the publication.
